# Protecting brain health: neurocognitive risks in sports players

**DOI:** 10.1016/j.ebiom.2025.105812

**Published:** 2025-06-11

**Authors:** eBioMedicine

Picture this: you are a proud parent standing on the side of a football field cheering for your team to score a goal; a quick turnaround, a couple of precise passes, and your little child comes from behind an opponent with a header to take the goalkeeper by surprise. GOAL!

This scenario is just one of the possible ways in which a player, willingly or unwillingly, might hit their head during a match. The incidence of head injuries is even higher in other kinds of contact sport, such as rugby or ice hockey: a 2024 report from the US National Center for Injury Prevention and Control found that 63.6% of reported traumatic brain injuries (TBIs) in children were the result of participation in sports, with 41.1% of these attributable to contact sports.

The association between TBIs and the risk of neurological disorders has been recognised in professional contact sport players, a group who are at high risk of sustaining repeated concussions. Concussion, a mild TBI, can cause short-term and long-term effects on brain function, physiology, and development. Chronic traumatic encephalopathy is a progressive neurodegenerative disease and has been diagnosed postmortem. In a 2022 retrospective cohort study published in the *Journal of Neurology, Neurosurgery & Psychiatry*, the mortality and rate of diagnosis of neurodegenerative disease in former rugby players was examined. Using electronic medical records, 412 male Scottish rugby players were matched by sex, age, and socioeconomic status to a control group of 1236 members of the general population. The study suggested that, although the average life expectancy of the players did not differ from the general population, the risk of developing a neurodegenerative disease was 2.67 times higher than the control group. A higher incidence of amyotrophic lateral sclerosis (ALS) in former contact sports players was reported in a 2023 analysis published in *Amyotrophic Lateral Sclerosis and Frontotemporal Degeneration*. The study, which used questionnaire data from 2247 male individuals (1326 patients and 921 controls) showed that practicing contact sport had 76% higher odds of an ALS diagnosis, particularly when combined with tobacco exposure. The researchers suggested that this increased risk might be related to the repetitive trauma to the head and cervical spine sustained during the practice of the sport. A 2023 systematic review in *eClinicalMedicine* reported elevated dementia risk in amateur and professional players of soccer, wrestling, and boxing, relative to the general.

Trauma induced by accelerations and decelerations of the head can, depending on the intensity, cause a different level of concussion that can be characterised by impairment of consciousness, reflex activities, and loss of memory, and can have long-term cognitive, motor, and mental health effects.

The repetitive aspect of sports head injuries is fundamental to understanding the mechanism of brain damage. In a February, 2025 publication in *Communications Biology*, the authors used a mouse model to compare the effect of a single mild concussion to the sensorimotor cortex with three concussions sustained 48 h apart. Motor behaviours did not differ between the groups of mice and no increase in cell death was detected. However, in the repetitive concussion group, learning deficits and sustained structural activation of microglia, accompanied by engulfment of excitatory synapses, were observed. In the central nervous system, microglia regulate homoeostasis and have an important role in injury repair. Microglia secrete neurotrophic factors and anti-inflammatory cytokine in tissue healing, but, autophagic dysregulation following a TBI can make the brain susceptible to secondary injury and neurodegeneration.

In September, 2024, WHO and FIFA launched a joint concussion awareness campaign to improve recognition of symptoms and provide medical guidance on how players can safely return to the game. Given the health risks associated with repeat concussions, it is important that the brain has fully recovered before resuming the sport. Some symptoms of concussion can take weeks to manifest, highlighting a need for diagnostic biomarkers to guide player management. In a study of 24 professional rugby players, published in *Brain Injury* in 2024, serum S100B was reported to temporarily increase following concussion. A prospective cohort study of Australian football players in *Neurology* found that serum glial fibrillary acidic protein measured within 24 h of suspected concussion had higher diagnostic value than symptom assessment alone.

Measures and interventions have been put in place to try to limit the risk of head injury during sport practice, including the 2024 ban introduced by the UK Football Association on heading for football players younger than 12 years. A 2025 publication in *BMJ Open Sport & Exercise Medicine* analysed videos from 60 rugby games to quantify the effect of a new rule that lowered the maximum legal height of tackling from the shoulders to the base of sternum, a move during which players are at highest risk of sustaining a concussion. The authors found that the new law led to a reduction in head-to-head and head-to-shoulder contact between players.

However, implementing more rules is not the only way to improve safety for players. In a May, 2025 publication in *eBioMedicine*, the authors propose that stabilising the head–neck–torso complex could help prevent the dangerous effects of repetitive head impacts related to heading in football. This crossover study investigated the effects of wearing a custom-made mouth guard during heading exercises in 18 amateur male players. Participants performed heading drills both with and without the mouth guard, while researchers measured the peak force of the neck flexor muscles, assessed cognitive function before and after the exercise, and recorded the cortical silent period (cSP) duration, as a measure of corticomotor inhibition. The results were very promising, showing that wearing the mouth guard led to a decrease in linear peak head acceleration and an increase in the peak force of neck muscles, suggesting enhanced physical protection. Physiologically, the study observed a significant decrease in the cSP, and cognitive assessments revealed a decreased change in memory performance.

While it is crucial to prioritise preventing and limiting the dangers of head injuries, it is equally important to acknowledge that accidents can happen in all physical activities, not just contact sports, and even a mild concussion can have life-changing consequences. Support for recovery is essential. A growing number of mobile health (mHealth) apps are being developed to aid individuals who have had head injuries. For example, an April, 2025 study in *JMIR Formative Research* described a mobile app designed to provide personalised exercise-based rehabilitation programmes for those recovering from concussions. At the end of a 2-week period, 23 participants rated the usability of the app through the mHealth App Usability Questionnaire, with 96% of participants self-reporting a wellbeing benefit. Flexible, at-home options such as this app highlight the importance of digital health solutions that can provide a user-friendly, evidence-based environment to boost patient confidence and enhance their recovery experience.

**T**here is a clear need to, whenever possible, prevent the occurrence of mild traumatic events in all sports, at all stages, and at all levels (from amateur to professional), while keeping intact the positive aspects of the sport experience. Sport clubs and associations should be responsible for their athlete's safety and introduce measures to prevent not only the occasional injuries that can occur on the field, but also the future negative outcomes on their health.

Biomedical research has an important role to play. From documenting the long-term risks of sports-related TBIs, revealing the underlying pathological mechanisms, and guiding the development of protective interventions, we must keep advocating for comprehensive research. All that should remain after a day on the sports field is the memory of an exciting game.

